# Public health events and economic growth in a neoclassical framework

**DOI:** 10.1186/s12889-024-19106-4

**Published:** 2024-06-28

**Authors:** Yunhao Wang, Yixuan Liu, Zhihan Peng, Zhaoyang Shang, Wei Gao

**Affiliations:** 1 Key Laboratory for Applied Statistics of MOE, School of Mathematics and Statistics, Northeast Normal University, Changchun, 130024 China; 2 School of Information Science and Technology, Northeast Normal University, Changchun, 130117 China; 3 Department of Computer Science, Duke University, Durham, 27708-0129 NC USA; 4 School of Economics and Management, Northeast Normal University, Changchun, 130117 China

**Keywords:** Public health event, Epidemic, Prevention and control intensity, Economic growth, Neoclassical growth model, C15, C62, E13, I18, J68, O11

## Abstract

Public health events (PHEs) have emerged as significant threats to human life, health, and economic growth. PHEs, such as COVID-19, have prompted a reevaluation for enhanced regular prevention and control (RPC). In this study, we focus on the core concept of prevention and control intensity (PCI), and establish a neoclassical economic growth model from the long-term and macro perspective to balance life protection and economic growth. The model construct the mechanism of PCI on economic growth through population dynamics and capital accumulation under the backdrop of RPC for PHEs. We find the conditions for PCI when the economy achieves steady state, and provides an algorithm establishing the optimal strategy that maximises per capita disposable income based on the optimal PCI and consumption. Simulation result quantifies an inverted U-shaped relationship between PCI and capital per capita, output per capita and consumption per capita in the steady state. The model suggests that, given the PHEs of inducing potential unemployment shock, it is worthwhile to combine the implementation of moderate PCI with coordinated policies of income distribution.

**Supplementary Information:**

The online version contains supplementary material available at 10.1186/s12889-024-19106-4.

## Highlights

• Building a Neoclassical economic growth model under public health events (PHEs).

• Model links regular prevention and control (RPC) to macroeconomic factors.

• Exploring mechanism of RPC for PHEs on economic growth.

• Appropriate prevention and control intensity can engender economic growth.

**Supplementary Information:**

The online version contains supplementary material available at 10.1186/s12889-024-19106-4.

## Introduction

Despite the lack of a universally-accepted definition of pandemic, it is clear that this pattern of disease has profoundly influenced human history from prehistory to the present day [1]. Throughout the ages, recurrent outbreaks of infectious diseases with the potential to become pandemics, such as pestilence, cholera, flu, AIDS, severe acute respiratory syndrome coronavirus (SARS-CoV), and Middle East respiratory syndrome coronavirus (MERS-CoV), have caused far more deaths than all wars, non-infectious diseases, and natural disasters combined [2]. The ongoing COVID-19 pandemic has been unique in its high contagiousness, plunging the global economy into the worst recession since World War II [3], and it is unlikely to be the last or most severe pandemic in the foreseeable future.

Extensive research has confirmed the significant impact of COVID-19 on the global economy. Statistical data and analysis reveal that the pandemic has caused a sharp decline in world GDP, with developing countries falling by approximately 2.5% and industrialized nations by around 1.8% [4]. Even after the pandemic subsides, it remains a challenge for economies to fully recover [5]. The International Labor Organization’s report [6] highlights that in 2020, the world lost nearly 9% of working hours, resulting in a significant reduction in full-time jobs and labor income equivalent to 4.4% of global GDP. Additionally, the pandemic has disrupted labor supply, increased production costs, and led to inflation, resulting in a substantial rise in unemployment rates [7, 8]. The World Investment Report [9] indicates that foreign direct investment globally declined by over a third during the pandemic, lower than that after the 2008 financial crisis. According to the World Development Report [10], the measures of dealing with COVID-19, such as mobility restrictions and quarantines, have triggered the most severe global economic crisis in over a century. This crisis has caused economic contraction in approximately 90% of countries, surpassing the two World Wars, the Great Depression of the 1930s, the emerging economy debt crises of the 1980s, and the 2007-2009 global financial crisis.

The far-reaching impact of COVID-19 has diverted scholars’ attention to the socioeconomic impact of infectious diseases, and a large number of studies have emerged [11]. Firstly, [12] concludes that “panic buying” is the key word of consumer behavior during COVID-19. Under the framework of the consumer behavior model, there are five macro forces: the COVID-19 pandemic and the technological, political-legal, economic, and socio-cultural environments. A logical consequence of their interaction is that the micro-environment (family, friends, acquaintances, society, the media, and companies) interacts with consumers through technology and digital media. Information from Social media, including advice from associates, product shortage perceptions, the COVID-19 spread, official announcements, and global news inspired panic buying, especially during lockdowns [13]. Besides, the personal and psychological characteristics of consumers determine how to interpret stimuli and make decisions, such as the impact of gender or age on panic purchases [14, 15], pandemic-induced negative psychological states and feelings [16, 17]. Secondly, the pandemic has brought unprecedented challenges to global corporations, strategic planning, human resource management, supply chain management, and buying interaction with consumers [18–20]. From the aspect of marketing strategies, companies during the pandemic will adjust to reduce various risks and increase benefits perceived by the consumer, thus encouraging online shopping, via digital technology to reduce physical contact [21]. In terms of size, the impact of COVID-19 on small and medium-sized enterprises is much more pronounced than that on large ones [22]. In addition, the pandemic has also had a comprehensive impact on the employment market [23] and the financial market [24], which has been proven to be an important source of market volatility.

In the emergent economics-epidemiological literature, a series of recent research have sought to identify optimal control policies based on the SIR framework [25–27]. Significantly, some scholars have utilized various theories to combine the SIR model and economics to analyze the macroeconomic impact of epidemics and the corresponding prevention and control policies (PCP), wherein individuals will be infected while engaging in consumption or work activities [28–30]. Drawing closer to the subject of those research, a few recent studies have constructed macroeconomic growth models involving epidemic factors, thereby exploring the long-term ramifications of the pandemic and the effects of alternative policy responses [31–33]. However, the structures like SIR or SEIR often mainly consider the short-term impact within a disease transmission cycle, and for the long-term impact of the economic system, one cycle of disease transmission may not be long enough. If the models of infectious diseases are simply extended to the long term, it is necessary to consider multiple infection cycles, which might be less convenient to the macroscopic analysis of the long-term impact of infectious diseases on the economy.

Given the urgency of PHEs such as COVID-19 and its longer-term and sustained transition [34], to prevent, control and manage that longer-term emergencies, a longer-term and economics-epidemiological framework is essential. Accordingly, countries need to adopt PCP under the background of regular prevention and control (RPC) for PHEs. In this article, we aim to develop a macro framework, responding to longer-term PHEs, to balance life protection and economic growth from a longer-term perspective. This framework can discuss the impact of PHEs on indicators such as population dynamics, population health, capital, and consumption from the perspective of steady economic growth, analyze the role of public health management in macroeconomic stabilization, and provide policy suggestions from an economic perspective for responding to PHEs. The main goal of this article is to propose the concept of prevention and control intensity (PCI) to measure PCP, and to establish an economics-epidemiological model, where PCI affects economic growth through population and capital, and find the optimal PCI from the perspective of economic growth, which can provide a reference for each economy to formulate more reasonable PCP and to respond to PHEs.

In this study, we introduce two key innovations that distinguish our model from existing macroeconomic growth theories based on epidemic models. Firstly, our proposed model takes a macroscopic and long-term perspective, allowing us to examine the impact of PHEs on economic growth without exploring the complexities of epidemic transmission dynamics. This broader approach provides a more comprehensive understanding of the ramifications of PHEs on the economy. Secondly, our model goes beyond merely considering the benefits of RPC for PHEs and incorporates the associated costs. By examining the transmission mechanism at the steady state, we propose an algorithm of the optimal PCI that is specifically designed to promote economic growth, which is constructive for formulating corresponding policies. This unique perspective recognizes that PCP not only directly affect population mobility but also influence capital accumulation through the expenditures allocated to these measures. As a result, our model offers valuable insights to the intricate relationship between PCP, capital accumulation, and ultimately, economic growth.

The ensuing sections of this paper are structured in the following manner: In “Methodology” section, we first explicate the concept of PCI in the context of RPC for PHEs. Subsequently, based on the relationship between PCI and its effect on the economy, a neoclassical economic growth framework is constructed, resolving for the optimal PCI. “RPC for PHEs and economic growth” section primarily focuses on the impact of RPC for PHEs of indefinite duration on economic growth and extends it to the case of non-permanent duration of PHEs. In “Numerical simulation” section, we calibrate a variety of parameters utilizing China’s experience in preventing and controlling the COVID-19 pandemic and conduct numerical simulations. “Further discussion beyond the model” section further discusses the impact mechanism of PHEs on economic growth. “Conclusion” section is the conclusion.

## Methodology

This section tries to construct a continuous-time neoclassical macroeconomic growth model under the background of RPC for PHEs (Unless otherwise stated, for any variable *Z* in the following text, it holds $Z=Z(t)$, meaning that the relevant variables in the model are functions of time). In economic modelling, output is usually viewed as a function encompassing labor and capital, known as the production function. Under the background of RPC for PHEs, human health is threatened, meaning PCP will affect population dynamics, public health expenditures, capital accumulation, and thus economic output. In this section, we firstly construct the relationship between PCI and the economy, particularly in terms of population structure and capital accumulation. Subsequently, a neoclassical economic growth model incorporating PCI is developed, with the equilibrium solution being derived from the per capita capital dynamics. Then, we discuss the determination of the optimal PCI and optimal marginal propensity to consume (MPC) through the optimization of disposable income.

### PHEs and PCI

#### RPC for PHEs

Since 2007, the World Health Organization has totally declared seven public health events of international concern, including the H1N1 influenza pandemic in 2009, the polio outbreak in 2014, the Ebola epidemic in West Africa in 2014, the Zika virus epidemic in 2016, the Ebola outbreak in Congo in 2018, the COVID-19 pandemic in 2019, and the Monkeypox epidemic in 2022. Without exception, all of these have been marked by the emergence and spread of infectious disease on a major scale. Consequently, the essence of the PHE hereinafter can be simply understood as the outbreak of infectious disease.

Following the occurence of a PHE , the government will initiate management and take measures to mitigate the impact. If the event is short-lived, it can be viewed as a short-term exogenous shock to the economy, allowing it gradually converge to its long-term steady state. Contrarily, it is assumed that the PHE cannot be completely resolved in this study, that is, the PHE will last for a long time, and the entire society will be forced to adopt a regular regime of PCP.

#### PCI and population segmentation

Let *N*(*t*) denote the total population of a society, with an exogenous natural rate growth of *n*, and $t \geqslant 0$ representing time. Supposing that a PHE outbreaks at time $t=0$ and ends at time *T*. Denote $\tau _t=1$ if $t \leqslant T$, and otherwise $\tau _t=0$. In the absence of a PHE, all individuals in the given society are part of the labor force. However, during the period of RPC, the population is divided into three distinct categories: normal labor force *L*(*t*), labor loss due to restricted mobility $R(t;\theta )$, and the infected people caused by the PHE $I(t;\theta )$. It is important to note that the sum of these three categories is equal to the number of the total population *N*(*t*).

1 $$\begin{aligned} L(t) + \tau _t R(t;\theta ) + \tau _t I(t;\theta ) = N(t), \; \theta \in [0, 1] \end{aligned}$$

where the PCI is defined as an exogenous parameter ranging from 0 to 1, which signifies the level of governmental intervention in a PHE. When $\theta =0$, the state adopts the most lenient PCP, allowing for unrestricted the movement of people. Whereas $\theta =1$ implies the most stringent PCP, with only the the most essential services being maintained in the city. For any PCI value between 0 and 1, we can reach them through various mobility restriction policies, for example a city with only 100 public transportation lines available for people to travel. Furthermore, let’s assume that the number of transportation lines has a linear relationship with population mobility. If all the lines are closed, the PCI will be close to 1, while if half of the traffic lines are stopped, the PCI will be approximately 0.5. Even if there exists a non-linear relationship between the number of transportation lines and population flow, there will still be corresponding mobility restriction policies due to its continuity. In practice, governments often implement different restrictive policies to deal with the PHEs. These policies may include cutting back traffic, closing restaurants, tourism and entertainment venues, extending holidays, and even implementing strict regional regulations such as city closures. However, all these can be evaluated by using a specific value of PCI. There are existing studies that assess the level of control associated with different policies [35, 36]. In other words, for any given value of PCI, there will be at least one corresponding restriction policy for implementation.

It is worth noting that there are some implicit assumptions. Firstly, under the background of RPC for PHEs, the disease that does not result in death or the mortality rate is too low to be considered. Typical examples include influenza or COVID-19, where death rate is gradually decreasing. This means that the number of the infected individuals can reflect the level of health protection. Furthermore, it is assumed that all individuals without PHEs in the system are laborers. The first reason is that the non-working population remains minorities compared with the working population, as considered in most classic macroeconomic models. Secondly, by ignoring the short-term disease transmission structure, Eq. (1) demonstrates that PCI is the sole factor influencing population composition. And the impact of other factors, such as population density, population labor structure (working and non-working population), age structure, urban-rural structure, etc., can be characterized by the function of PCI, the population and its parameters. Thirdly, this structure can be easily generalized to non-working populations due to its per-capita form.

Consequently, PCI directly restricts the labor flow and, indirectly, the number of normal labor force by influencing the number of individuals infected by the PHE.

#### Expenditure on PCP

To regulate the governance of PHEs, the government needs to make certain fiscal expenditures, referred to as total expenditures on prevention and control (TEPC) for the management of PHEs, which can be divided into three distinct categories based on the division of the population: the basic expenditure on prevention and control (BEPC), the expenditure on restricting population mobility (ERPM), and the expenditure on patient treatment (EPT).

Denoted as $X_1$, BEPC is a public policy intended for all individuals, comprising measures such as vaccination, construction of isolation facilities, and nucleic acid testing to effectively tackle PHEs. This expenditure is an increasing function of PCI, in other words, PCI is directly proportional to the level of BEPC, i.e.,

2 $$\begin{aligned} X_1 = X_1(N(t);\theta ), \quad \frac{\partial X_1}{\partial \theta } > 0. \end{aligned}$$

To facilitate calculation without sacrificing generality, it can be assumed that the per capita BEPC is proportional to PCI for each unit of population. Consequently, $X_1 = N(t) h_1 \theta$, where $h_1$ represents the per capita BEPC when $\theta =1$, that is, the maximum per capita BEPC.

To regulate a PHE, it is essential to restrict the mobility of certain populations, thereby necessitating ERPM. ERPM, denoted as $X_2$, is the social costs incurred as a result of isolation and blockade measures taken against the relevant personnel, encompassing isolation expenses and the sustenance of control personnel. Intuitively, the higher the PCI, the more significant is the labor loss due to the restriction of mobility, thus leading to an increase in this expenditure, i.e.,

3 $$\begin{aligned} X_2 = X_2(R(t;\theta )), \quad \frac{\partial X_2}{\partial R}> 0, \frac{\partial R}{\partial \theta }> 0, \frac{\partial X_2}{\partial \theta }=\frac{\partial X_2}{\partial R}\frac{\partial R}{\partial \theta }>0. \end{aligned}$$

To further simplify, we assume that the cost of restricting the movement of one unit of labor force (denoted as $h_2$) is uniform, then $X_2=h_2 R(t;\theta )$.

Furthermore, PHEs can lead to the presence of patients, and containment of its propagation through the treatment of patients is essential for the regulation of such occurrences. The EPT, namely the expenditure incurred for the care of infected people, is denoted as $X_3$, which has a positive correlation with the number of patients. In order to capture the transmission mechanism of PCI, this study does not employ an infectious disease model to estimate the daily number of patients, but instead adopts a generalized but practical macro perspective for analysis. Specifically, the high PCI will impede the spread of the PHE, thereby decreasing the number of patients, enhancing the health of the population, and diminishing the EPT caused by the PHE, namely,

4 $$\begin{aligned} X_3 = X_3(I(t;\theta )), \quad \frac{\partial X_3}{\partial I} > 0, \frac{\partial I}{\partial \theta }< 0, \frac{\partial X_3}{\partial \theta }=\frac{\partial X_3}{\partial I}\frac{\partial I}{\partial \theta }<0. \end{aligned}$$

Similarly, assuming that the required EPT per patient (denoted as $h_3$) is the same, we have $X_3=h_3 I(t;\theta )$.

**Fig. 1:**
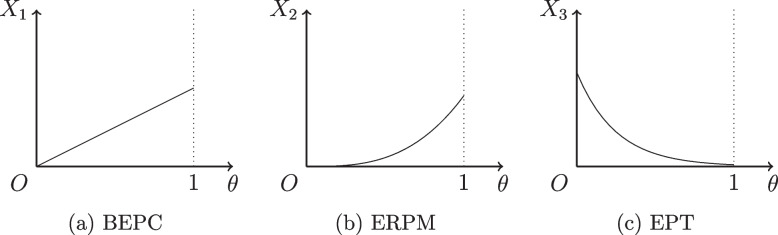
Expenditure function of PCI

For any given time *t*, a range of potential expenditure functions are shown in Fig. 1. As demonstrated in Fig. 1a, BEPC is proportional to PCI. Figure 1b illustrates that ERPM grows exponentially in accordance with PCI, with the number of normal labor force limited in their mobility rising as PCI rises, thus leading to a corresponding increase in ERPM. Moreover, the higher the PCI, the more rapid the growth of the labor loss, which in turn result in a rise of the marginal ERPM. Figure 1c shows that EPT decreases exponentially as PCI increases, with the diminution of population mobility and the spread of the PHE slowing down, the number of patients gradually decreases, thereby causing a decrease in the number of patients and a concomitant decrease in EPT. Simultaneously, the higher the PCI, the slower the rate of patients decreases, thus inducing a reduction in the marginal EPT.

The TEPC, denoted as *X*, is the sum of the three types of expenditures mentioned above, i.e.,

5 $$\begin{aligned} X=X(t;\theta ){} & {} =X_1(t;\theta ) + X_2(R(t;\theta ))+X_3(I(t;\theta )) \nonumber \\{} & {} = N(t)h_1 \theta + h_2 R(t;\theta )+ h_3 I(t;\theta ). \end{aligned}$$

**Fig. 2:**
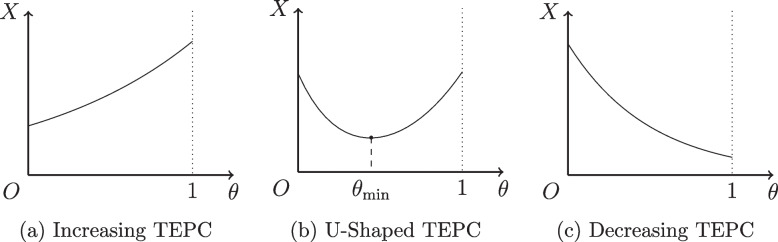
Function of TEPC

Figure 2 serves to illustrate the three potential forms of the TEPC. Figure 2a demonstrates the TEPC is positively correlated to the PCI, while Fig. 2c exhibits a decreasing function of the TEPC. In practice, the most probable case, however, is shown in Fig. 2b, where the TEPC is a U-shaped function of the PCI, thereby producing a decrease-then-increase-type function of the TEPC with a minimum point, $\theta _{\min }$.

### Economic growth model with PCI

#### Production function

Consider a production function with classical assumptions, including constant returns to scale in production, diminishing marginal productivity and Inada conditions, etc., which only focuses on capital and labor force rather than technological advancement (more details about technological progress are given in Supplementary Material 3):

6 $$\begin{aligned} Y(t)=F(K(t), L(t)), \end{aligned}$$

where *Y*(*t*), *K*(*t*) and *L*(*t*) represent for total output, capital and normal labor force, respectively. A typical production function is the Cobb-Douglas form, which can be expressed as

7 $$\begin{aligned} Y(t)=F(K(t), L(t))=B K(t)^{\alpha }L(t)^{1-\alpha }, \end{aligned}$$

where *B* is a constant representing the level of technology, and $\alpha$ represents the output elasticity of capital.

#### Capital dynamics

Due to the impact of the PHE, the total output will be allocated to consumption, investment and TEPC. Therefore, investment equivalent to the net savings which remain after subtracting consumption, TEPC and the depreciation of existing capital stock, i.e.,

8 $$\begin{aligned} \dot{K}(t)=Y(t) - C(t) - \tau _t X(t;\theta ) - \delta K(t), \end{aligned}$$

where *C*(*t*) denotes consumption, $\delta$ represents the rate of capital depreciation, and $\dot{K}(t)$ is the change rate of capital or net investment. To simplify, we assume that consumption is an exogenous linear function of disposable income, which can be expressed as

9 $$\begin{aligned} C(t) = (1-\tau _t)\beta _1 Y(t) + \tau _t \beta _2 [Y(t)-X(t;\theta )], \end{aligned}$$

where $0< \beta _1, \beta _2 < 1$ is the marginal propensity to consume (MPC) respectively under normal circumstances and the outbreak of PHEs, during which a portion of the total output needs to be allocated for EPCs. Therefore, disposable income can be represented as $Y(t) - X(t; \theta )$. Now, the capital dynamics equation can be rewritten as

10 $$\begin{aligned} \dot{K}(t)= (1-\beta _1+\tau _t\beta _1-\tau _t\beta _2)Y(t) - (1-\beta _2) \tau _t X(t;\theta )] - \delta K(t). \end{aligned}$$

Given that the optimal policy will include the MPC, simplifying the constant MPC allows us to ignore the intermediate path and directly analyze the impact of changing the MPC [37].

#### Per capita form

Equations (1), (5), (6) and (10) constitute an economic growth model with PCI, where both sides of the equations are divided by *N*(*t*) to obtain the per capita form.

Equation (1) can be transformed into the per capita form by assuming that the three types of population are proportional to the total population, i.e.,

11 $$\begin{aligned} l(t)+\tau _tr(t;\theta )+\tau _ti(t;\theta )=1, \end{aligned}$$

where $l=L(t)/N(t)$, $r=R(t;\theta )/N(t)$, and $i=I(t;\theta )/N(t)$ represent the proportions of normal labor force, the labor loss and the infected people to the total population, respectively. Now Eq. (1) is transformed to represent the proportion of each population group.

Formula (5) can be converted into the per capita function for the TEPC,

12 $$\begin{aligned} x=x(t;\theta ) = x_1(t;\theta ) + x_2(r(t;\theta )) + x_3(i(t;\theta )), \end{aligned}$$

where

13 $$\begin{aligned} x(t;\theta )=X(t;\theta )/N(t), \end{aligned}$$

14 $$\begin{aligned} x_1(t;\theta )=X_1(N(t);\theta )/N(t), \end{aligned}$$

15 $$\begin{aligned} x_2(r(t;\theta ))=X_2(R(t;\theta ))/N(t)=X_2(N(t)r(t;\theta ))/N(t), \end{aligned}$$

16 $$\begin{aligned} x_3(i(t;\theta ))=X_3(I(t;\theta ))/N(t)=X_3(N(t)i(t;\theta ))/N(t) \end{aligned}$$

represent the per capita TEPC, BEPC, ERPM and EPT, respectively.

Equation (6) can be transformed into per capita production function

17 $$\begin{aligned} y(t)=F(k(t), l(t)), \end{aligned}$$

where $y(t)=Y(t)/N(t)$ and $k(t)=K(t)/N(t)$ represent per capita output and capital, respectively.

Equation (10) can be transformed into per capita capital dynamics. Using $\dot{K}(t)=\dot{N}(t)k(t)+N(t)\dot{k}(t)$ yields

18 $$\begin{aligned} \dot{k}(t)=(1-\beta _1+\tau _t\beta _1-\tau _t\beta _2)F(k(t), l(t)) - (n+\delta ) k(t) - (1-\beta _2) \tau _t x(t;\theta ), \end{aligned}$$

where $n=\dot{N}(t)/N(t)$ denotes the natural growth rate of population.

## RPC for PHEs and economic growth

In this section, we discuss three cases: the absence of PHEs, an infinite-term PHE, and a finite-term PHE. First, we briefly discuss the situation where no PHE exists. Then, we focus on the impact of RPC for PHEs on economic growth in the context of an infinite-term PHE, and extend this analysis to the case of a finite-term PHE.

### Normal case: complete absence of PHEs

Firstly, consider the case when $t > T$, that is, when there is no PHE. Under normal circumstances, no PCP are needed, and the entire population is the labor force, i.e., $l(t) = 1$. In this situation, the model degenerates into the classical Solow model, and the capital dynamic equation is as follows:

19 $$\begin{aligned} \dot{k}(t)=(1-\beta _1)F(k(t),1) - (n+\delta ) k(t). \end{aligned}$$

Setting it to zero, we can obtain the steady-state per capita capital $k_{0}^*$, which satisfies:

20 $$\begin{aligned} (1-\beta _1)F(k_{0}^*, 1) = (n+\delta ) k_{0}^*. \end{aligned}$$

Now, let’s examine the convergence of any *k*(*t*) to $k_{0}^*$. A first-order Taylor approximation of $\dot{k}(t)$ around $k = k_{0}^*$ yields:

21 $$\begin{aligned} \dot{k} \approx -\lambda _0[k(t)-k_{0}^*], \end{aligned}$$

where

22 $$\begin{aligned} \lambda _0{} & {} = -\frac{\partial \dot{k}}{ \partial k}\left| _{k=k_0^*} =(n+\delta )-(1-\beta _1)\frac{\partial F(k, 1)}{ \partial k}\right| _{k=k_0^*} \nonumber \\{} & {} =(n+\delta )-(n+\delta )\frac{ k_{0}^*}{F(k_{0}^*, 1)}\left.\frac{\partial F(k, 1)}{ \partial k}\right| _{k=k_0^*} =[1-\alpha _{k}(k_{0}^*,1)](n+\delta ). \end{aligned}$$

The speed at which *k*(*t*) converges to its value $k_0^*$ on the balanced growth path is given by the convergence rate. Due to constant returns to scale,

$$\alpha _{k}(k_{0}^*,1)=\frac{ k_{0}^*}{F(k_{0}^*, 1)}\frac{\partial F(k, 1)}{ \partial k}\left| _{k=k_0^*} = \frac{\partial k}{\partial F(k,l)} \frac{\partial F(k, l)}{\partial k} =\alpha \right.$$

is precisely the output elasticity of capital. Equation (22) implies that near the balanced growth path, the speed at which *k*(*t*) converges to $k_{0}^*$ is directly proportional to the distance between them. Furthermore, we can calculate:

23 $$\begin{aligned} k(t) \approx k_{0}^* + e^{-\lambda _0 (t-T)}[k(T)-k_{0}^*], \end{aligned}$$

where *k*(*T*) is the per capita capital at time *T*. This means that for any two moments $t'>t'' \geqslant T$ satisfying $k(t')<k_{0}^*$ and $k(t'')<k_{0}^*$, then $k(t')>k(t'')$, and the time required for the per capita capital to converge to the steady state $k_{0}^*$ at time $t'$ is shorter.

When $0 \leqslant t \leqslant T$, $\tau _t=1$, the capital dynamic equation becomes:

24 $$\begin{aligned} \dot{k}(t)=(1-\beta _2)F(k(t), l(t)) - (n+\delta ) k(t) - (1-\beta _2)x(t;\theta ). \end{aligned}$$

In this case, the economic system is a dynamic process. We will discuss two scenarios separately: indefinite continuation of PHEs ($T = \infty$) and non-long-term persistence of PHEs ($T < \infty$).

### An infinite-term PHE

#### Steady state

If the disease continues indefinitely, i.e., when $T=\infty$, there may be an equilibrium point for per capita capital. At equilibrium, where any exogenous given PCI is fixed, per capita capital stock remains constant, as evidenced by the rate of change of per capita capital $\dot{k}$ satisfying $\dot{k}=0$. More specifically, since $\theta$ is exogenously given, the proportions of labor loss $r=r(\theta )$, the proportion of infected people $i=i(\theta )$ and the per capita TEPC $x=x(\theta )$, are all independent of the passage of time at equilibrium. Correspondingly, the proportion of normal labor force to the overall population $l=1-r-i$ also remains constant irrespective of time. Therefore, the per capita capital at equilibrium, denoted as $k^*$, satisfies

25 $$\begin{aligned} (1-\beta _2)F(k^*, l) = (n+\delta ) k^* + (1-\beta _2)x. \end{aligned}$$

As opposed to the Solow’s model [38], the break-even investment curve, the right-hand side of Eq. (25) is not coincident with the origin due to the presence of the TEPC, instead being shifted upwards. Analysis in Supplementary Material 2 shows that the steady state of the economy is existing with PCI in its feasible range, denoted as $\theta \in [\theta _L, \theta _U]$, $0 \leqslant \theta _L < \theta _U \leqslant 1$. And more details about the steady state and the balanced growth path (BGP) are also given in Supplementary Material 2.

#### Optimal policy: Optimizing PCI and MPC

Having previously considered the steady state of the economy when subject to a predetermined level of PCI, in this subsection, we will explore the effects of varying levels of PCI and MPC upon the steady state.

##### Optimal PCI

It is noted that PCI is fixed in the above analysis. Now there is a natural question that of which value of PCI is the optimal. Indeed, the various level of PCI will make different economic goals. To define the optimal PCI, the goals might be set as maximizing per capita output, minimizing per capita TEPC, and maximizing per capita disposable income. As previously elucidated, the per capita capital at the steady state can be obtained from Eq. (25) when PCI is given, thus rendering $k^*$ a function of $\theta$. Consequently, Eq. (25) holds for any given $\theta$. Therefore, taking the derivative of both sides of Eq. (25) concerning $\theta$ yields an equality:

26 $$\begin{aligned} (1-\beta _2)\left[ \frac{\partial F}{\partial k^*} \frac{\partial k^*}{\partial \theta } + \frac{\partial F}{\partial l} \frac{\partial l}{\partial \theta } \right] =(n+\delta )\frac{\partial k^*}{\partial \theta } + (1-\beta _2) \frac{\partial x}{\partial \theta }, \end{aligned}$$

where $\frac{\partial l}{\partial \theta }=-\frac{\partial r}{\partial \theta }-\frac{\partial i}{\partial \theta }$ is the rate of change of the proportion of normal labor force to total population with respect to the PCI.

Equation (26) encapsulates the influence of modifications in PCI on corresponding variables. By means of a comparative static analysis, we shall discuss the optimal PCI when the economy arrives at a steady state. Intuitively, the optimal PCI ought to maximize the per capita output, $F(k^*,l)$, in the steady state and concurrently minimize the per capita TEPC, *x*. The following Theorem 1 provides the conditions for achieving both objectives simultaneously, with its proof in Supplementary Material 1.

##### Theorem 1

(Maximizing per capita output and minimizing per capita TEPC). Assuming the economy is on the steady state described by Eq. (25). When other conditions remain unchanged, if there exists PCI, $\theta =\theta _{both}^*$, such that both

27 $$\begin{aligned} \frac{\partial k^*}{\partial \theta } = \frac{\partial x}{\partial \theta } = 0 \end{aligned}$$

and

28 $$\begin{aligned} \frac{\partial x_1}{\partial \theta } = (h_3 - h_2) \frac{\partial r}{\partial \theta } \end{aligned}$$

are satisfied, then the economy can simultaneously achieve the maximization of the per capita output and the minimization of the per capita TEPC.

Theorem 1 shows that, if the optimal PCI $\theta _{both}^*$ exactly makes both Eqs. (27) and (28) hold, the per capita capital in the steady state will be maximized, while the per capita TEPC will be simultaneously minimized. However, it is of great possibility that the function both $k^*(\theta )$ and $x(\theta )$ cannot reach extreme points at the same time, which means both attaining objectives simultaneously is an ideal state.

Therefore, a second-best solution or an alternative approach is to find PCI that maximizes per capita disposable income (the difference between per capita output and per capita TEPC). At this point, the optimal problem is changed as

29 $$\begin{aligned} \underset{\theta }{\textrm{max}}\, y_d = y - x =F(k^*, l) - x. \end{aligned}$$

Note that *y*, $k^*$, *l* and *x* are all functions of $\theta$ in the equation.

##### Theorem 2

(Maximizing per capita disposable income). Assuming the economy is on the steady state characterized by Eq. (25), and with all other conditions held constant, the solution to the equation

30 $$\begin{aligned} \frac{\partial k^*}{\partial \theta } = 0, \end{aligned}$$

denoted as $\theta _d^*$, represents the PCI that maximizes the per capita disposable income in the economy.

The proof of Theorem 2 is given in Supplementary Material 1. Compared to $\theta _{both}^*$, which both maximizes per capita output and minimizes per capita TEPC, $\theta _{d}^*$ that maximizes disposable income is only required to satisfy Eq. (30), thus yielding the maximum disposable income and per capita capital. It is noteworthy that the optimal levels of per capita output and TEPC may not be attained with $\theta _{d}^*$. Nevertheless, since this goal of maximizing disposable income is always achievable, it is defined as the optimal PCI that the government can implement.

##### Optimal MPC

Apart from PCI, MPC is another parameter that affects per capita capital and output. For any given PCI, the steady state per capita capital in Eq. (25) can be viewed as a function of MPC. Following the Solow model, a golden rule is ascertained for per capita capital stock in the context of RPC for a PHE, that is, for any given PCI, finding the optimal MPC to maximize per capita consumption at the steady state,

31 $$\begin{aligned} \underset{\beta _2}{\textrm{max}}\ c= \beta _2 (y^* - x), \; \text {for any given } \theta , \end{aligned}$$

where *c* is the per capita consumption corresponding to the steady state; $x=x(\theta )$ is the per capita TEPC for any given $\theta$; $k^*$ is the per capita capital; $l=1-r(\theta )-i(\theta )$ is the proportion of normal labor force, and $y^*=F(k^*,l)$ is the per capita output. Denoted the solution to Formula (31) as $\beta _2^*$, the following Theorem 3 shows the condition of $\beta _2^*$ which maximizes the per capita consumption, with its proof in Supplementary Material 1.

##### Theorem 3

Suppose the economy is on the steady state characterized by Eq. (25). Holding all other conditions constant, the optimal MPC that maximizes consumption in the economy is, $\beta _2^*$, satisfies the condition

32 $$\begin{aligned} \beta _2 \frac{\partial y^*}{\partial \beta _2} + y^*-x=0 \end{aligned}$$

or

33 $$\begin{aligned} \beta _2(1-\beta _2) \frac{\partial k^*}{\partial \beta _2} + k^*=0. \end{aligned}$$

Theorem 3 indicates that, for any given PCI, there exists optimal MPC that maximizes the per capita consumption, thereby achieving the golden rule level of capital stock. It is worth noting that, since PCI is exogenously given, thus the optimal MPC is also a function of the PCI.

##### Optimal policy

In the previous text, the optimal PCI and MPC are discussed separately. Indeed, they both can be regarded as optimal policy in the model because they both can measure the intensity of policy in the RPC for PHEs. In addition to PCI, the parameter most affected by policy is the MPC. Generally, the government can influence the proportion of output used for investment (i.e., the savings rate), and thereby affect the MPC, by adjusting government revenue and expenditure and levying taxes on savings and investment. Therefore, $(\theta , \beta _2)$ constitute the policy parameters in the model. To select the optimal policy, a numerical algorithm is provided based on the optimization of PCI and MPC, with more details as shown in Algorithm 1.

**Figure:**
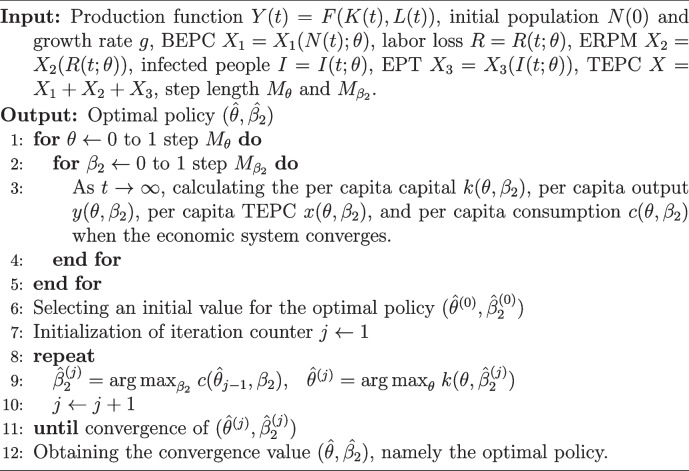
**Algorithm 1** Numerical algorithm for selecting the optimal policy

### A finite-term PHE

When $t>T$, the PHE disappears, and the normal state is restored. Suppose the per capita capital stock at the end of the PHE is *k*(*T*), then at this time *k*(*T*) converges to $k_0^*$. The larger *k*(*T*) is, the shorter the time required for convergence to $k_0^*$. Therefore, during the PHEs, the PCI $\theta$ should be chosen to maximize *k*(*T*). During the sudden PHE, i.e., $0 \leqslant t \leqslant T$, let $k_{\theta }(t)$ denote the per capita capital at time *t* when the PCI is $\theta$, and let $\theta _{T}^*$ be the optimal PCI when the PHE lasts only until time *T*. The optimal PCI should maximize the per capita capital at time *T*, i.e.,

34 $$\begin{aligned} \theta _{T}^*=\arg \max _{\theta } k_{\theta }(T). \end{aligned}$$

Let the steady-state per capita capital corresponding to the optimal PCI $\theta _{d}^*$ in Theorem 2 be denoted as $k_{d}^*$. Obviously, when $T \rightarrow \infty$, $k_{\theta }(t)$ should converge to $k_{d}^*$. As shown in Eq. (25), changes in $\theta$ will result in changes in the corresponding steady-state per capita capital $k_{\theta }^*$; therefore, when the end time *T* of the PHE is known, the above optimization problem can be solved using numerical methods. However, it is challenging to know the end time *T* of the PHE in advance. Therefore, the next best option is to choose the optimal PCI $\theta _{d}^*$ during the PHE. The following discussion focuses on the economic growth situation when the PCI $\theta ^*_d$ is chosen, and the PHE lasts for a limited time. For any known $\theta$, perform a first-order Taylor expansion at $k_{\theta }^*$:

35 $$\begin{aligned} k_{\theta }(T) \approx k_{\theta }^* + e^{-\lambda (\theta ) T}[k(0)-k_{\theta }^*], \end{aligned}$$

where *k*(0) is the per capita capital at time 0; the growth rate is:

36 $$\begin{aligned} \lambda (\theta ){} & {} = \left.{-\frac{\partial \dot{k}}{ \partial k}}\right|_{{k=k}_{\theta}^{*}} =\left.(n+\delta )-(1-\beta _2)\frac{\partial F(k^*, l)}{ \partial k}\right|_{k=k_{\theta }^*} \nonumber \\{} & {} =(n+\delta )\left[1-\frac{k^*}{F(k^*, l)-x}\frac{\partial F(k^*, l)}{ \partial k}\right]_{k=k_{\theta }^*} \nonumber \\{} & {} =(n+\delta )\left[1-\frac{F(k^*, l)}{F(k^*, l)-x}\alpha _{k}(k^*,l)\right]_{k=k_{\theta }^*}, \end{aligned}$$

which is also a function of $\theta$, and

37 $$\begin{aligned} \alpha _{k}(k^*,l)=\frac{ k^*}{F(k^*, l)}\left.\frac{\partial F(k, l)}{ \partial k}\right| _{k=k_{\theta }^*}=\alpha . \end{aligned}$$

By performing a first-order expansion of $k_{\theta }(T)$ at $\theta ^*_d$, we obtain:

38 $$\begin{aligned} k_{\theta }(T){} & {} \approx k_{\theta ^*_d}^* + e^{-\lambda (\theta ^*_d) T}\left[k(0)-k_{\theta ^*_d}^*\right] \nonumber \\{} & {} + \left[ \frac{\partial k_{\theta }^*}{\partial \theta } \left| _{\theta =\theta ^*_d} - Te^{-\lambda (\theta ^*_d) T}\frac{\partial \lambda }{\partial \theta }\left| _{\theta =\theta ^*_d} + e^{-\lambda (\theta ^*_d) T}\frac{\partial k_\theta ^*}{\partial \theta } \right| \right. _{\theta =\theta ^*_d}\right] (\theta -\theta ^*_d) \nonumber \\{} & {} \left. = k_{\theta ^*_d}^* + e^{-\lambda (\theta ^*_d) T}[k(0)-k_{\theta ^*_d}^*] - Te^{-\lambda (\theta ^*_d) T}\frac{\partial \lambda }{\partial \theta }\right| _{\theta =\theta ^*_d}(\theta -\theta ^*_d), \end{aligned}$$

where

39 $$\begin{aligned} \left.\frac{\partial \lambda }{\partial \theta }\right| _{\theta =\theta ^*_d}{} & {} =-(n+\delta )\left[ \frac{F(k_{\theta ^*_d}^*,l)\frac{\partial x}{\partial \theta }-\left[ \frac{\partial F}{\partial k} \frac{\partial k}{\partial \theta } + \frac{\partial F}{\partial l} \frac{\partial l}{\partial \theta } \right] x}{(F(k_{\theta ^*_d}^*,l)-x)^2}\right] _{\theta =\theta ^*_d} \nonumber \\{} & {} \left. = -(n+\delta )\frac{{\partial x}/{\partial \theta }}{F(k_{\theta ^*_d}^*,l)-x} \right| _{\theta =\theta ^*_d}. \end{aligned}$$

Note that in the above equation, $\theta =\theta ^*_d$ is written outside the parentheses because *k*, *l*, and *x* are all functions of $\theta$.

Considering the deviation between $k_{\theta }(T)$ and $k_{\theta ^*_d}^*$,

40 $$\begin{aligned} \Delta = e^{-\lambda (\theta ^*_d) T}\left[k(0)-k_{\theta ^*_d}^*\right] - Te^{-\lambda (\theta ^*_d) T}\left.\frac{\partial \lambda }{\partial \theta }\right| _{\theta =\theta ^*_d}(\theta -\theta ^*_d), \end{aligned}$$

as *T* increases, the exponential decay occurs. This indicates that for an unknown PHE in practice, although it is hard to know its end time, the optimal PCI $\theta ^*_d$ can be also selected based on Theorem 2. And when the PHE ends, there is no need for prevention and control.

## Numerical simulation

In this section, a series of numerical simulations are performed, based upon the model previously described and the parameters set by China’s experience in the RPC for COVID-19. Initially, the relevant functions and parameters in the model are established. Next, a simulation is made under three different cases, where Case 1 is normal, Case 2 is a indefinite PHE (including three sub-cases of the TEPC, and Case 3 is a short-term PHE. Finally, a comparison and analysis of simulation results are given.

### Functions and parameters settings

The settings of relevant functions and parameters in the model are shown in Tables 1 and 2, respectively.

**Table 1:** Functions settings

Functions	Specific settings
Production function	$Y(t)=B K(t)^{\alpha } L(t)^{1 - \alpha }$
Capital dynamics	$\dot{K}(t) = Y(t) - C(t) - X(t;\theta ) - \delta K(t)$
Consumption	$C(t) = \beta _2 \left[ Y(t) - X(t;\theta )\right]$
BEPC	$X_1(N(t);\theta ) = N(t) h_1 \theta$
Labor loss	$R(t;\theta )= r_m N(t) \theta ^3$
ERPM	$X_2(R(t;\theta )) = h_2 R(t;\theta )$
Infected people	$I(t;\theta )= i_m N(t) \exp (-\tau \theta )$
EPT	$X_3(I(t;\theta )) = h_3 I(t;\theta )$

Note: As presented in Table 1, the function of labor loss, $R(t;\theta )$, is set as a cubic function, with $r_m$ denoting the proportion of labor loss to total population when the PCI equals to 1. Additionally, The function of infected people, $I(t;\theta )$, is established as an exponential function, with $i_m$ representing the proportion of infected people to total population when the PCI equals to 1, while $\tau$ measures the decreasing rate of infected people brought about by PCI

**Table 2:** Parameters settings

Parameters	Values	Sources
$\alpha$	0.5	[39]
$\delta$	0.05/365	[39]
*n*	0.0053/365	[40]
*N*(0)	1	Normalized to 1
*K*(0)	482000	[41]
*B*	0.40	Setting
$r_m$	0.95	Setting
$i_m$	0.15	Setting^a^
$\tau$	3	Setting
$(h_1,h_2,h_3)$^b^	(20,10,300)	U-shaped TEPC (Case 2.1 and Case 3)
	(20,10,5)	Increase-type TEPC (Case 2.2)
	(20,10,2000)	Decrease-type TEPC (Case 2.3)

^a^Note 1: Table 2 presents Omicron as an instance, with an estimated basic reproduction number of the infectious disease of 15-25, an incubation period of 3-5 days, a recovery period of 7-10 days, and an assumed immune escape period of 90 days [42]. The above parameters are approximations based on average values. Utilizing SIRS model, the proportion of infected people in the steady state can be calculated as $\dfrac{(1-1/20)/90}{1/(4+8.5)+1/90}=11.59\%$. Considering the reality of some level of isolation and social distancing measures, it is reasonable to set infected proportion as $i_m=15\%$ when the PCI equals to 0

^b^Note 2: As $(h_1,h_2,h_3)$ takes the values of (20,10,300), (20,10,5) and (20,10,2000), the functions of the TEPC correspond to the three cases in Fig. 2

### Simulation results

#### Case 1: Normal case

**Fig. 3:**
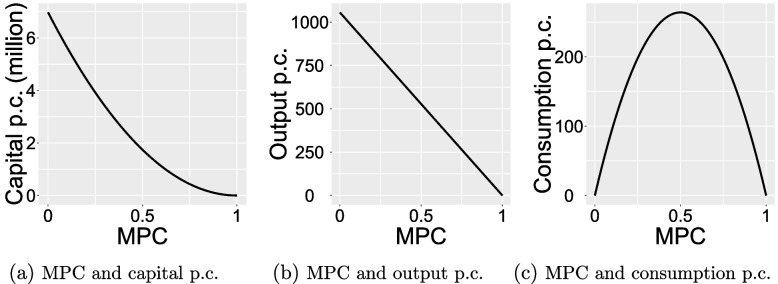
MPC and per capita (p.c.) capital, output and consumption under normal case

Under the normal case, i.e., without a PHE, the indicators like population and capital will increase at the growth rate of stead state. To facilitate comparison with the cases of PHEs, Fig. 3 illustrates the relationship between the MPC and per capita capital, output, and consumption at the steady state under normal conditions. It is apparent that the MPC is inversely proportional to per capita capital and output. As for per capita consumption, its peak is attained when the MPC is 0.5 with the per capita capital, output and consumption at the steady state reaching 1.74 million, 528.02 and 264.01, respectively. At this point, the annual growth rate of total output is approximately 5.5%.

#### Case 2: An infinite-term PHE

**Fig. 4:**
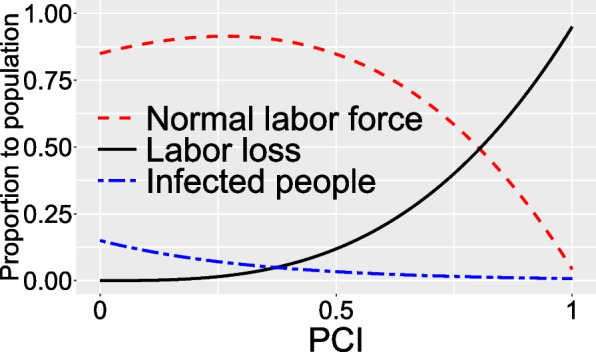
Population dynamics under different PCI at the steady state

Compared with Case 1, the PHE last for an infinite-term in Case 2. Given that there are three forms of TEPC (shown in Fig. 2), the three sub-cases will be discussed, marked as Case 2.1, 2.2 and 2.3, corresponding to U-shaped, increase-type, and decrease-type TEPC, respectively. At the steady state, the impact of PCI on population dynamics is shown in Fig. 4. When PCI is zero, the labor loss is zero, but infected people to the total population will reach 15%, resulting in increased EPT and reduced disposable income. As PCI initially adds, although the proportion of labor loss increases exponentially, it can also quickly reduce the number of infections, allowing the proportion of normal labor to increase. As the PCI rises to 0.27, the proportion of normal labor reaches a peak of 91.46%. As PCI continuously increases, the proportion of normal labor affected by labor loss goes up and exceeds the number of infections, resulting in a corresponding reduction in the normal labor.

Population dynamics lead to economic dynamics. To further analyze the numerical discrepancies of variables at the steady state under different values of PCI, we set the MPC at 0.47, 0.49, and 0.40 under three different cases and adjust PCI. The corresponding variables under varying PCI are provided in Table 3, which reveals an inverted U-shaped relationship both between PCI and capital per capita, output per capita and consumption per capita at the steady state, no matter which kind of sub-cases.

**Table 3:**
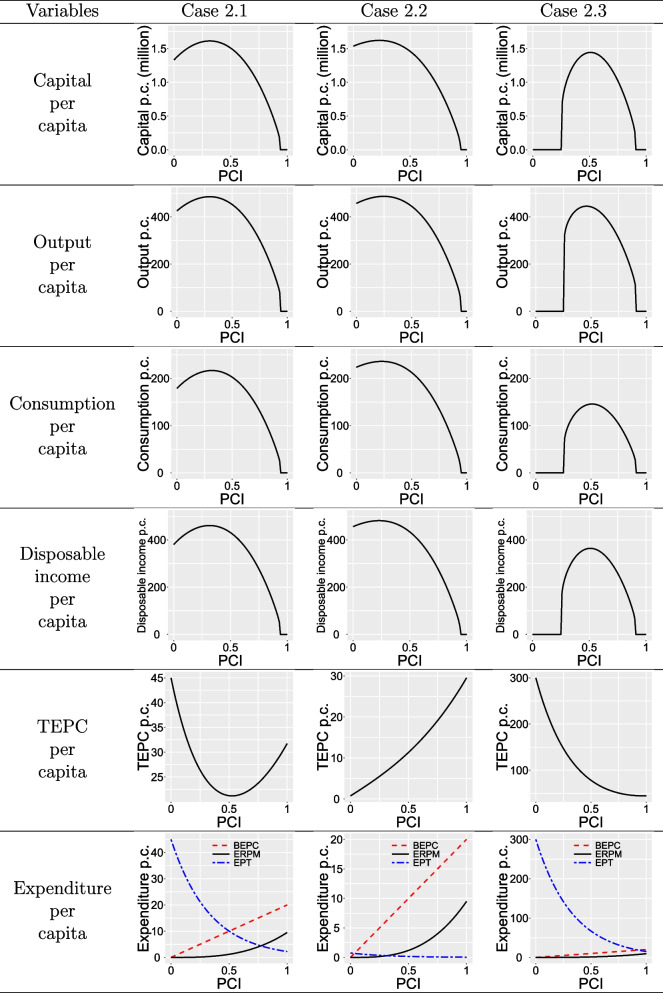
Economic dynamics under different PCI at the steady state

The U-shaped TEPC hypothesis may be more realistic because too high or too low PCI will lead to higher TEPC. For Case 2.1, although the optimal PCI can not minimize TEPC, it will increase the number of normal labor, causing output growth exceeding the increase in TEPC, thereby increasing per capita disposable income in the steady state. In Case 2.2 and Case 2.3, no matter how TEPC increases or decreases due to PCI, the optimal PCI will not drop to the bottom of TEPC. At this point, the optimal PCI economically balances the number of infected people with the normal labor and maximizes per capita disposable income, despite facing higher expenditures and capital accumulation challenges. In short, too high or too low PCI may hinder capital accumulation and reduce output in the steady state. Additionally, when PCI approaches 1, even though there are only a few infected people, a larger number of people are restricted, resulting in a shortage of normal labor, and per capita output is insufficient to cover per capita EPT and capital depreciation, causing insufficient capital accumulation and thus an economic recession. In particular, when PCI approaches 0 in Case 2.3, a large number of infected people and the resulting high treatment costs also hinder the normal accumulation of capital, thereby leading to economic recession.

In addition, the optimal PCI with three different types of TEPC is 0.32, 0.23, and 0.51, respectively. This indicates that the optimal PCI tends to be oriented towards a decrease in TEPC to some degree. Despite the fact that an increase in PCI may result in a corresponding rise in the TEPC while leading to a substantial diminishment of the labor force in the same time, a moderate degree of PCI remains an indispensable requirement.

Similar to analyzing economic dynamics under different PCI, we fix PCI at 0.32, 0.23 and 0.51, and vary the marginal propensity. The economic dynamics can be observed in Table 4. Per capita capital, output, and disposable income display a monotonic decrease with increasing MPC. Conversely, per capita consumption follows an inverted U-shape, peaking at MPC values of 0.47, 0.49, and 0.40, with respective values of 461.01, 481.60, and 364.32, similar to the Solow model. In the context of PHE, the MPC should not be excessively high (e.g., in Case 2.1 exceeding 0.9 and Case 2.3 exceeding 0.65), as this could impede capital accumulation, resulting in economic stagnation. To facilitate a deeper understanding of the impact of PHEs, specific values of the variables at the steady state for Case 1 and Cases 2.1-2.3 during PHEs are summarized in Table 5.

**Table 4:**
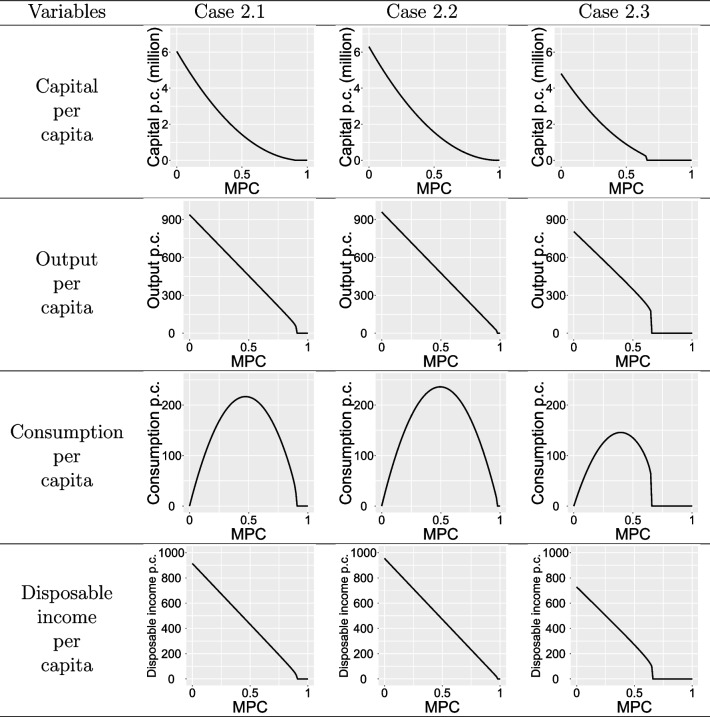
Economic dynamics under different MPCs at the steady state

**Table 5:** Variables in the steady state under the optimal policies in the normal case and the cases during PHEs

Variables	Case 1	Case 2.1	Case 2.2	Case 2.3
Optimal PCI	/	0.32	0.23	0.51
Optimal MPC	0.5	0.47	0.49	0.40
Capital per capita (million)	1.74	1.61	1.62	1.44
Output per capita	528.02	484.95	486.69	440.75
Consumption per capita	264.01	216.67	235.98	145.73
TEPC per capita	0	23.94	5.09	76.42
Disposable income per capita	528.02	461.01	481.60	364.32
Proportion of normal labor force	100%	91.14%	91.32%	84.15%
Proportion of labor loss	0	3.11%	1.16%	12.60%
Proportion of infected people	0	5.74%	7.52%	3.25%

#### Case 3: A finite-term PHE

In Case 3, the PHE will last only for a finite term *T*, which is set as $T=0, 3, 6, 9, 12, 15$. During PHEs, the setting of the optimal parameters are the same as Case 2.1, i.e, setting PCI as 0.32 and MPC as 0.47). To show various sub-cases where savings rate changes after the PHE ends, the MPC after PHEs is set as 0.4, 0.45 and 0.5, respectively. Table 6 shows the dynamics of per capita capital and per capita output. Generally speaking, the longer the duration of PHE, the further the growth curves of per capita capital and per capita output shift to the right, and the greater the impact on per capita capital accumulation and per capita output. However, once the PHE ends, its impact on the economy will gradually diminish over time.

**Table 6:**
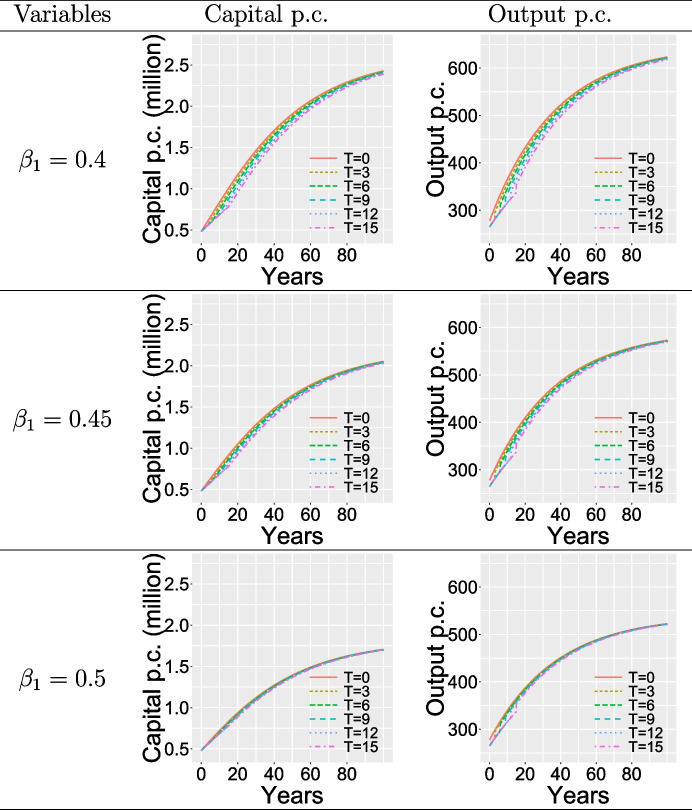
Finite-term PHEs under different MPCs

## Further discussion beyond the model

Having previously established a neoclassical economic growth model based on PCI and elucidated the impact mechanism of PHEs on the economic growth, as well as determining the optimal PCI and MPC, we now proceed to further discuss the impact mechanisms of PHEs. The first subsection explores the impacts of PHEs on total factor productivity (TFP) and price levels, while in “TFP and price mechanism” subsection, these impacts will be examined. In “Policies in PHEs” subsection, we will examine the various economic policies adopted by different countries and regions in response to PHEs. Finally, we paraphrase economics in PHEs.

### PHEs in a short period

This paper mainly investigates the macroeconomic dynamics under the background of long-term PHEs. However, some PHEs last only for a short period from outbreak to termination. A PHE can be considered a short-term severe shock at the initial stage of its outbreak. Unlike RPC for long-term PHEs, in general, targeted and reasonable preventive and control measures and effective drugs for curing the disease cannot be provided within a short period after the outbreak of a PHE. At this time, the treatment of infected patients requires extremely high costs. For instance, within one year after the outbreak of the COVID-19 pandemic, the median treatment cost for COVID-19 patients in the United States reached $1,772 ﻿[43]. If considered in the TEPC function, the TEPC would be decreasing, but with a very high EPT. At this point, governments often adopt high PCI to reduce the further spread of infectious diseases. Although implementing high-intensity control measures affects economic development, it can minimize human losses. However, if the spread of the infectious disease cannot be effectively contained in a short time, the PHE will turn from a short-term shock into a long-term event.

Furthermore, if PHEs are caused by diseases with mild symptoms, or there are targeted drugs available for treatment, making the treatment cost of patients approximate to the treatment cost under normalized prevention and control, the TEPC is approximately U-shaped. Although it is a short-term PHE, its optimal control intensity is still close to the optimal control intensity of long-term management. In other words, when a PHE meeting these conditions occurs, measures similar to long-term prevention and control can still be adopted to cope with it in the short term.

### TFP and price mechanism

As the aim of this research is to incorporate and analyze RPC for PHEs on the framework of neoclassical economic growth, the PHE mainly affected the economy through two pathways in our proposed model based on PCI: population and capital. Nevertheless, PHEs can also influence the economy through TFP and price mechanisms in reality, which shall be addressed in the following discussion.

#### Impact of PHEs on TFP

As a pivotal measure of technological advancement and resource allocation efficiency, TFP is widely regarded as the driving force behind sustainable economic growth. Previous studies have demonstrated that PHEs can cause significant and, in some cases, irreversible reduction of TFP. For example, the outbreak of SARS epidemic in 2003 led to a notable 3.12%-5.81% decline in TFP for Chinese industrial enterprises [44], while the COVID-19 pandemic in 2020 inflicted a permanent deceleration on the growth rate of TFP in the United States [45].

From the perspective of mechanism, the impact of PHEs on TFP may be analyzed through the following main pathways: Firstly, such events may directly affect urban transportation systems, leading to a decrease in production efficiency and subsequently a decline in TFP [46]. Secondly, the school closure policies implemented by various countries to contain the spread of viruses in PHEs may affect children and young people, resulting in a slowdown in human capital accumulation [47]. Furthermore, PHEs themselves and the control policies they necessitate can impact the global supply chain system, consequently reducing TFP [48, 49]. At last, the policy measures taken by governments to control PHEs may result in resource mismatches and efficiency losses, ultimately leading to a decline in TFP [50].

#### Price mechanism in PHEs

PHEs have far-reaching consequences beyond their impact on TFP. They also affect market supply and demand relationships, and can influence the economy through price mechanisms. For example, the COVID-19 pandemic, as an international PHE, has exerted a profound influence on the economic system by affecting the prices of various goods, encompassing food, energy, and real estate.

From the perspective of supply shock, the pandemic has resulted in a scarcity of daily necessities, thus leading to upward movements for many BLS food price indexes, particularly for meat products, which have had a large price increase [51]. Furthermore, the pandemic has significantly reduced the transportation of goods, resulting in increased transportation costs and varying degrees of price increases [52]. From a demand shock perspective, the pandemic has led to a decrease in energy demand, resulting in a decrease in energy prices. As a driving force in the modern economic society, changes in energy prices, particularly oil prices, have a significant impact on the economy [53].

Due to the price war between Saudi Arabia and Russia, the Ukraine conflict, and the suspension of industrial machinery and transportation activities during PHEs, some international energy prices, such as oil, have suffered severe shocks, resulting in fluctuations in international trade, increased uncertainty in economic policies of various countries, and even international balance of payment crises [54]. Additionally, increases in energy prices, such as oil and coal, will increase production costs, reduce consumer spending, and decrease corporate investment [55], leading to a decline in gross domestic product and even the emergence of malignant inflation [56].

Moreover, PHEs have an impact on housing prices. Empirical studies have demonstrated that areas with confirmed cases of the disease have experienced a significant decrease in housing prices (2.47% in China [57] and 1.26% in Australia [58]). From the perspective of population density distribution, the pandemic has driven the demand for housing to shift to suburban areas, resulting in significant differences in housing rental prices between central cities and suburbs [59].

In summary, PHEs can result in fluctuations in price levels, thereby causing alterations in consumer choices and preferences, as well as producer costs and investments, ultimately having profound effects on the economy.

### Policies in PHEs

To cope with PHEs, governments have primarily adopted two types of policies: PCP for public health and economic policies, which will be discussed partly below.

#### PCP in PHEs

According to the OxCGRT dataset [35], most countries adopted strict PCP to counteract COVID-19 with average annual PCI above 0.5. This signifies a global consensus on the pivotal role of PCP in RPC for PHEs and safeguarding lives. Simultaneously, due to disparate population densities, healthcare infrastructures, and socio-economic circumstances, coupled with varying timelines of infectious outbreak onset, the specific PCP were employed distinctly among the countries. Consequently, the effects of these PCP also diverge significantly. Certain countries have opted for high-PCI strategies, such as China, Singapore and South Korea. They implemented strict PCP in the early 2020, such as case tracing, quarantine, isolation, even city lockdown, swiftly curtailing the spread of the virus and thereby efficaciously protecting lives [60]. Conversely, others have either embraced relatively lenient PCP or delayed their implementation, such as Canada [61] and the United States [62], whose PCP during COVID-19 might have a greater negative impact on their citizens’ health.

As mentioned above, COVID-19 has had a huge impact on the world economy. In terms of heterogeneity, due to different economic factors such as economic development, economic structure and consumption structure, the impact of the COVID-19 pandemic on the economies of various countries also varies. Some countries like China and Singapore [60], adopted strict PCP in the early pandemic, which reduced labor losses and struggled to maintain positive economic growth rates, and most countries began to experience economic decline after being hit by the pandemic. Unfortunately, these facts do not help researchers analyze the role of different PCP. An intriguing question about PHEs is which PCI (or corresponding PCP) can ensure economic growth. A theoretical framework proposed in this study suggests that the impact of PCI on the economy follows an inverted U-shaped curve, and its peak might correspond to vastly different levels of PCI across countries due to their unique circumstances. Intuitively, to assess whether Country A, which has chosen high PCI in reality, should have opted for lower PCI, it is vital to estimate the counterfactual outcome (an inherently unobservable outcome in reality). Then, a more appropriate conclusion can be discerned by comparing the real outcome and the counterfactual outcome. It is imperative to note that the outcomes from Country B with lower PCI, cannot be directly shifted to Country A due to heterogeneity. Therefore, it is worth to further discuss the impact of PCP on economy during PHEs in the future when faced with the observed data.

In practice, it is difficult to comprehensively manage PHEs. From the perspective of public administration, the containment measures in response to PHEs should not be “one-size-fits-all”. Instead, considerations should be given to the specific conditions of a country, including its economic status, demographic structure, healthcare infrastructure, and available public resources. Additionally, an appropriate containment strategy requires a relative balance between protecting lives and fostering economic development. Moreover, the disparities in managing PHEs among various countries also pose challenges to global governance. The diverse PCP employed by countries result in varying outcomes in combating the PHEs. The global transmission of these PHEs, in turn, challenges the efficacy of PCP, stressing the imperative need for robust international collaboration and coordination. The success of global governance hinges on adequately respecting the distinctiveness and diversity of each country, which means that countries should not only be vigilant about the global spread of PHEs but also fully recognize and accept the differences among them.

#### Economic policies in PHEs

To mitigate the economic impact of PHEs, in addition to implementing PCP aimed at protecting lives, various countries often enact policies to maintain the economic stability. The following will discuss income distribution, social welfare, fiscal policies, and monetary policies.

##### Income distribution and social welfare in PHEs

In addition to expansion of the economic “cake”, the distribution of income, or how the “cake” is divided, is also a crucial issue in RPC for PHEs. The exacerbation of income inequality can impede the GDP growth rate [63]. Recent research has demonstrated that a significant portion of the population in countries with elevated levels of income inequality experiences instability and endures challenges, such as inadequate housing, disease and pollution, which culminate in higher mortality rates during the pandemic when compared to countries with lower levels of income inequality [64].

In response to the emergencies of PHEs, countries and regions over the world have implemented varying measures and income distribution policies, aimed at reducing the deleterious impact of such events on social and economic activities. Developed countries such as EU member states [65], Australia [66], and developing countries such as China [67], have taken a series of policies and measures throughout the pandemic to maintain the living standards of low-income groups and avoid exacerbation of income inequality. These policies have included the provision of additional wage subsidies and welfare support, as well as enhancement of a range of social policies, including social insurance, social employment, and social welfare, all designed to counteract the exacerbation of income inequality brought about by the pandemic.

In addition, when governments endeavor to implement measures to address income inequality, they must also ensure that the corresponding policies are capable of achieving the expected results. During the COVID-19 pandemic, the US government implemented a series of relief bills and policies, providing a large amount of relief funds and policies, which provided substantial assistance to the middle and lower-income groups. Regrettably, due to insufficient government oversight of financial activities, many low-income individuals invested their relief funds in the stock market, resulting in a significant influx of capital into the hands of the wealthy, who already possess more than 80% of the stocks, resulting in low-income people being unable to receive social welfare in practice, thereby exacerbating the wealth gap in the United States [68]. Meanwhile, South Africa implemented lockdown policies to fight against the pandemic but failed to adopt appropriate transfer payment policies, which had a severe impact on low-income families where some workers had lower levels of education, ultimately exacerbating income inequality indirectly [69].

In summary, it is incumbent upon governments to promptly implement temporary policies and effective social welfare distribution measures to improve income distribution during PHEs. This must be achieved while maintaining an equilibrium between efficiency and equity in order to ensure expeditious and optimal economic advancement.

##### Fiscal and monetary policy in PHEs

Against the backdrop of RPC for PHEs, the government’s fiscal, monetary, and employment policies play a crucial role in ensuring steady and healthy economic development. Reasonable fiscal and monetary policies can alleviate the impact of PHEs. The South African government, for instance, has implemented a series of policies, including providing credit, loan extensions and tax relief, and offering unemployment insurance while enforcing lockdown measures to address the pandemic, with a view to stimulating the market economy [70]. During the COVID-19 pandemic, the Federal Reserve adopted an unlimited quantitative easing policy to assuage the panic in the financial markets by injecting a large amount of funds into the market through currency issuance, thereby stabilizing the economy in the short term [71]. However, this unconventional policy has led to historically high levels of assets and liabilities in the United States, exerting a significant impact on the economies and financial markets of emerging economies such as China, and casting a great deal of uncertainty on the development of the US economy [72]. The European Central Bank took prompt monetary policy action at the onset of the pandemic by implementing special bonds and conducting long-term refinancing operations with other commercial banks to absorb possible term risk caused by price fluctuations in the market, thus stabilizing the financial market and providing adequate liquidity for capital, making a decisive contribution to the relaxation of the financial situation in the euro zone [73].

In light of the COVID-19 pandemic, the Chinese government has implemented an active fiscal policy and a prudent monetary policy to carry out macroeconomic regulation and stabilize the economy, while maintaining the welfare of its citizens. During COVID-19, the Chinese government has intensified its efforts to reduce taxes and fees for small and medium-sized enterprises, individual businesses, and crucial industries such as manufacturing, capitalizing on the full guarantee capacity of fiscal policy to steadfastly safeguard the “three guarantees” (basic livelihood, wages, and operational continuity) [74]. In terms of monetary policy, the People’s Bank of China has consistently lowered the reserve requirement ratio for deposits, skillfully leveraging the traction and driving role of the policy of re-lending and re-discounting to provide precise financial services for PCP [75]. The government has emphasized that “active fiscal policies should be strengthened to improve efficiency, prudent monetary policies should be precise and powerful”. As the COVID-19 pandemic has progressed from an abrupt PHE to a long-term coexistence with sporadic outbreaks in different regions, relevant policies have also been continuously tilted towards public risk management and stable sustainability.

### Economic perspective in PHEs

Responding to PHEs is a comprehensive field that requires support from multiple disciplines. Economic factors are closely related to public health. The fundamental goals of public health management are health promotion and disease prevention. In the short term, public health management can be carried out regardless of economic costs. However, when facing long-term public health events, without sufficient resources, effective public health management is unattainable. Therefore, PRC for PHEs will inevitably imply economic stability, or say, the advanced goal of public health management. This means that it is very necessary to examine public health management from an economic perspective, which can provide economic support for responding to PHEs. Economics plays an important role in PHEs for the following reasons.

Firstly, PHEs can not only cause resources to become scarce, but also lead to the reallocation of resources, such as medicines and medical equipment, and the reallocation of public expenditures. Since economics studies the allocation of scarce resources, it can play an important role in PHEs. Although the fundamental goal of public health management is to protect life, the better the medical conditions in economically developed countries, the higher the degree of life protection and health promotion [76]. Therefore, in practice, the economic status of countries is the key to public health management. Discussing PHEs from an economic perspective can help us understand how to allocate limited resources most effectively to ensure the operation of the public health system and other industries.

Secondly, PHEs will have a broad impact on the population and capital in the economic system, changing the mode of production and consumption, then in turn affecting public health management. For example, an outbreak may result in factory shutdowns, less income, reduced consumption, etc. Evaluating the impact of PHEs from an economic perspective can play an important role in formulating public health and economic policies. For example, when deciding whether to lock down or whether to grant personal subsidies and corporate assistance, the economic impact needs to be considered.

To sum up, economics provides a new perspective on responding to PHEs. Different economic bases often lead to different public health responses. When formulating economic policies, governments need to consider both public health and economic stability. The specific content and implemented methods of these policies may vary from country to country, depending on their economic conditions, policy objectives, and feasibility.

## Conclusion

In this study, against the backdrop of RPC for PHEs, we first present a neoclassical macroeconomic growth framework based on population classification and PCI. Furthermore, we discuss the optimal PCI and MPC based on the steady state of the model, and obtain the corresponding optimal conditions, along with an algorithm for finding the optimal policy. Additionally, the numerical simulation results demonstrate that the model is highly interpretable and can offer guidance and policy recommendations for RPC for PHEs. Finally, further discussion is made beyond our proposed model. The main conclusions of our research are as follows:

(a) Our proposed model neglects many secondary factors. Nonetheless, it effectively elucidates the transmission mechanism of the impact of RPC for PHEs on economic growth from a macro and long-term perspective. The model demonstrates that an appropriate range of PCI can engender economic growth, and there exists PCI that maximizes per capita disposable income. Simulation results quantify an inverted U-shaped relationship between PCI and capital per capita, output per capita and consumption per capita in the steady state.

(b) PCI and MPC are two important parameters affected by policies in the model. For any PCI, there exists optimal MPC that maximizes consumption. Meanwhile, even if PHEs are short-term, the policies based on long-term PHEs are still effective.

(c) When confronted with PHEs that may result in a substantial number of unemployment, the implementation of moderate PCI combined with coordinated income distribution policies can effectively minimize the impact of such events on the economy, thereby guaranteeing sustained economic growth in the long run.

Economic growth is the advanced goal of RPC for PHEs, and it is necessary to examine public health management from an economic perspective. The study chooses a relatively simple neoclassical economic growth model, which mainly combined the Solow model with PHEs, and ignores further discussion of growth factors in the economic growth. In our model, the end date of the PHEs is exogenous, and all workers survive after infection. In the future research, the above assumptions can be relaxed. For example, the end date of the PHEs can be determined endogenously by the number of the infected and the probability of infection, and deaths can be considered through a transition structure. Furthermore, more factors about economic growth can be deeply considered and more complete and systematic economic models can be established to discuss the economic impact of PHEs. For example, we can consider the endogenization of consumption or a time-varying MPC (like Ramsey model), the population structure (age structure, labor structure, urban-rural structure, etc.) and intergenerational transmission behavior in the economic system (like overlapping generations model), the endogenization of technological progress (like endogenous growth model or R &D model), and dynamic stochastic general equilibrium model, etc.

Meanwhile, this research mainly treats the management of PHEs as an exogenous policy, while ignoring that the factors like labor and capital in economy will in turn affect PHEs and their management. In fact, due to the intricacy of the public management system and the economic system, there should be a dynamic interaction among them. Enhancing the integration of economics into PHEs should be poised to emerge as a future trend. Additionally, PHEs have different effects on various industrial sectors in the direction and intensity. In the future, more detailed discussions at the industrial level can be considered to improve the effectiveness of public health management. Therefore, considering more details on the public management in the public health system will in turn help to further clarify the transmission path and mechanism of the impact of PHEs on the economy.

## Supplementary information

Supplementary Material 1.

Supplementary Material 2.

Supplementary Material 3.

## Data Availability

All data and materials generated or analysed during this study are included in this published article.
