# Supplementary material for: Public health events and economic growth in a neoclassical framework
Source: BMC Public Health. 2024 Jun 28;24:1724. doi: 10.1186/s12889-024-19106-4 (PMC11539698; doi:10.1186/s12889-024-19106-4)
Supplement: Supplementary file 1 — Supplementary Material 1. [file 12889_2024_19106_MOESM1_ESM.pdf]

Proof of the theorems

Proof of Theorem 1 Firstly, the goal is the maximization of the per capita output by adjusting PCI, that is,

$$\max_{\theta} y = F(k^*, l). \tag{A.1}$$

The corresponding first-order condition is

$$\frac{\partial y}{\partial \theta} = \left[\frac{\partial F}{\partial k^*}\frac{\partial k^*}{\partial \theta} + \frac{\partial F}{\partial l}\frac{\partial l}{\partial \theta}\right] = 0. \tag{A.2}$$

Substituting Equation (A.2) into (26), we obtain

$$(n+\delta)\frac{\partial k^*}{\partial \theta} = -(1-\beta_2)\frac{\partial x}{\partial \theta}. \tag{A.3}$$

The solution to the above equation, $\theta = \theta_1^*$, represents the optimal PCI that maximizes output.

Next, we adjust the PCI to minimize the TEPC, x, that is,

$$\min_{\theta} x(\theta) = x_1(\theta) + h_2 r(\theta) + h_3 i(\theta). \tag{A.4}$$

The corresponding first-order condition is

$$\frac{\partial x}{\partial \theta} = \frac{\partial x_1}{\partial \theta} + h_2\frac{\partial r}{\partial \theta} + h_3\frac{\partial i}{\partial \theta} = 0. \tag{A.5}$$

The solution to Equation (A.5), $\theta = \theta_2^*$, is the PCI while minimizing the TEPC.

Different objectives may correspond to different optimal PCIs. Therefore, the issue worth considering is whether maximizing output and minimizing expenditures can be both satisfied.

If Equation (A.3) and (A.5) can be satisfied simultaneously, i.e., there exists an optimal PCI $\theta_{both}^* = \theta_1^* = \theta_2^*$ that satisfies Equation (27), then substituting Equation (27) into (A.2), we get

$$\left.\frac{\partial l}{\partial \theta}\right|_{\theta=\theta_{both}^*} = 0. \tag{A.6}$$

That is to say, the normal labor force in the economy is also maximized. Then, with

$$\frac{\partial l}{\partial \theta} + \frac{\partial r}{\partial \theta} + \frac{\partial i}{\partial \theta} = 0, \tag{A.7}$$

we obtain

$$\left.\frac{\partial r}{\partial \theta}\right|_{\theta=\theta_{both}^*} = -\left.\frac{\partial i}{\partial \theta}\right|_{\theta=\theta_{both}^*}. \tag{A.8}$$

At this point, the first-order condition (A.5) becomes Equation (28). Only when the optimal PCI $\theta_{both}^* = \theta_1^* = \theta_2^*$ satisfies (28), can the goals of maximizing output and minimizing expenditure be achieved simultaneously.

Proof of Theorem 2 The corresponding first-order condition of Equation (29) is

$$\frac{\partial y}{\partial \theta} - \frac{\partial x}{\partial \theta} = \left[\frac{\partial F}{\partial k^*}\frac{\partial k^*}{\partial \theta} + \frac{\partial F}{\partial l}\frac{\partial l}{\partial \theta}\right] - \frac{\partial x}{\partial \theta} = 0. \tag{A.9}$$

Substituting Equation (A.9) into (26), Equation (30) holds.

Proof of Theorem 3 The Lagrangian function for the optimization problem (31) subject to constraint (25) is

$$\mathscr{L} = \beta_2(y^* - x) + \mu[(1-\beta_2)(y^* - x) - (n+\delta)k^*], \tag{A.10}$$

where μ is the Lagrangian multiplier. Let

$$\frac{\partial \mathscr{L}}{\partial \beta_2} = (y^* - x) + \beta_2\frac{\partial y^*}{\partial \beta_2} + \mu[-(y^* - x) + (1-\beta_2)\frac{\partial y^*}{\partial \beta_2} - (n+\delta)\frac{\partial k^*}{\partial \beta_2}] = 0, \tag{A.11}$$

then

$$(y^* - x)(1-\mu) + [\beta_2 + \mu(1-\beta_2)]\frac{\partial y^*}{\partial \beta_2} - \mu(n+\delta)\frac{\partial k^*}{\partial \beta_2} = 0. \tag{A.12}$$

Additionally, with

$$\frac{\partial \mathscr{L}}{\partial \mu} = (1-\beta_2)(y^* - x) - (n+\delta)k^* = 0, \tag{A.13}$$

and taking the derivative with respect to β_2, we can see that

$$-(y^* - x) + (1-\beta_2)\frac{\partial y^*}{\partial \beta_2} - (n+\delta)\frac{\partial k^*}{\partial \beta_2} = 0. \tag{A.14}$$

Multiplying both sides of the equation by $-\mu$ and adding it to Equation (A.12), we obtain Equation (32). Then from Equation (A.13), we know that

$$y^* - x = \frac{n+\delta}{1-\beta_2}k^*. \tag{A.15}$$

Taking the derivative of both sides with respect to β_2, we get

$$\frac{\partial y^*}{\partial \beta_2} = (n+\delta)\left[\frac{1}{1-\beta_2}\frac{\partial k^*}{\partial \beta_2} + \frac{k^*}{(1-\beta_2)^2}\right]. \tag{A.16}$$

Substituting the above two equations into Equation (32) yields (33).
