# Supplementary material for: Public health events and economic growth in a neoclassical framework
Source: BMC Public Health. 2024 Jun 28;24:1724. doi: 10.1186/s12889-024-19106-4 (PMC11539698; doi:10.1186/s12889-024-19106-4)
Supplement: Supplementary file 2 — Supplementary Material 2. [file 12889_2024_19106_MOESM2_ESM.pdf]

Note on an infinite-term PHE

Existence and stability of solutions

In this subsection, we consider the equilibrium solution for per capita capital in the presence of a predetermined level of the PCI, as well as the stability of the solution. For any given PCI, Figure 1 illustrates the influence of different levels of TEPC on the equilibrium per capita capital. The existence and stability of the equilibrium solution under each case are analyzed below.

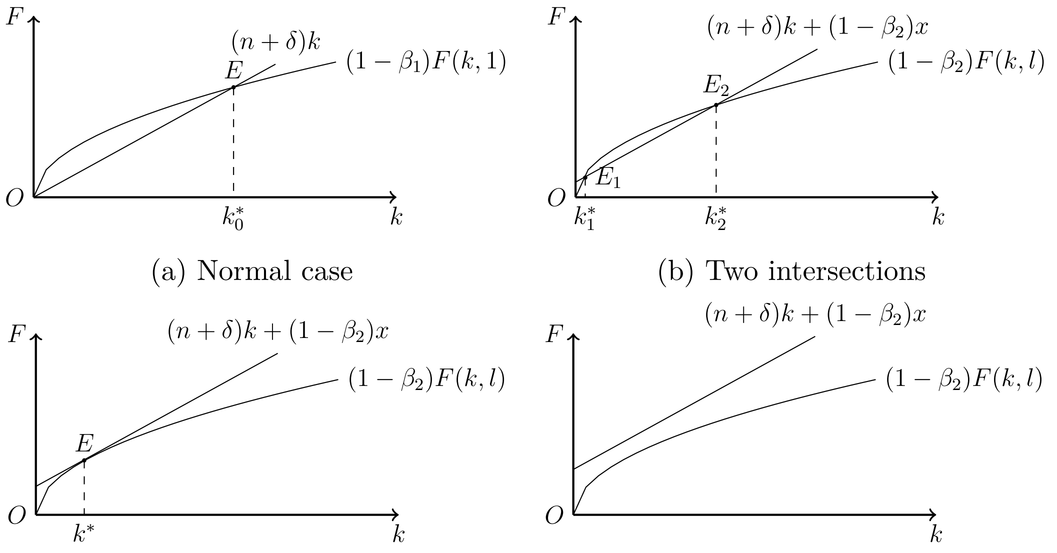

(a) Normal case

(b) Two intersections

(c) One tangent intersection

(d) No intersection

FIGURE 1 The impact of different levels of TEPC on the steady state of per capita capital

Figure 1(a) depicts a normal case without a PHE and the corresponding per capita TEPC is always zero. All individuals in the economy are normal labor force. The actual investment curve intersects with the break-even investment curve at the point E, corresponding to an equilibrium per capita capital level of k^*, which is a stable solution. If $k > k_1^*$, according to Equation (18), $\dot{k} > 0$, and k will increase to k^*; conversely, when $k < k_1^*$, $\dot{k} < 0$, and k will decrease to k^*.

Figure 1(b) depicts a case of RPC for a PHE with a relatively low per capita TEPC. The intersection of the actual investment curve and the break-even investment curve occurs at two points, E_1 and E_2, corresponding to the per capita capital levels of k_1^* and k_2^*, respectively, both of which are equilibrium solutions, yet only k_2^* is a stable solution with the logic of Figure 1(a). For the equilibrium solution $k = k_1^*$, if $k > k_1^*$, resulting in $\dot{k} > 0$, then k will increase to k_2^*. Conversely, if $k < k_1^*$, causing $\dot{k} < 0$, then k will decrease to 0, thus demonstrating that the point E_1 is merely the "knife-edge equilibrium" (Harrod, 1939; Domar, 1946).

Figure 1(c) portrays a case when the per capita TEPC is moderately high. In this case, the actual investment curve intersects with the break-even investment curve at the point E, corresponding to the per capita capital level of k^*, which is the equilibrium solution at the tangency point. Since the actual investment is

invariably smaller than or equal to the break-even investment, i.e., $\dot{k} \leqslant 0$, when $k > k^*$, k will decrease to k^*; conversely, when $k < k^*$, k will decrease to 0.

Figure 1(d) illustrates the case of a extremely high per capita TEPC, where the actual investment does not intersect with the break-even investment, thus indicating an economic decline. As the actual investment is less than the break-even investment, namely $\dot{k} < 0$, k will gradually dwindle to 0.

In summary, to prevent the economy from suffering long-term decline, Figure 1(b) presents a more realistic case, which dictates the PCI should be kept within a feasible range (called the feasible range of PCI), denoted as $\theta \in [\theta_L, \theta_U]$, where θ_L and θ_U ($0 \leqslant \theta_L < \theta_U \leqslant 1$) are respectively the lower and upper bounds of the feasible range of PCI. If the PCI is outside of its range, i.e., $\theta \in [0, \theta_L)$ or $\theta \in (\theta_U, 1]$, the break-even investment will be higher than the actual investment, causing the economy to suffer a decrease in per capita capital, gradually coverging to 0. Meanwhile, to ensure that per capita capital always converges to the steady state k_2^* instead of k_1^* in Figure 1(b), it is imperative that the initial per capita capital $k(0) > k_1^*$.

BGP

According to the above analysis, in the absence of technological progress, as long as the PCI is in its feasible range and the initial per capita capital $k(0) > k_1^*$, complying with the Figure 1(b), the economy will always converges to the BGP.

On the BGP, the growth rate of per capita capital is zero, implying that per capita capital remains constant (i.e., capital deepening is zero). Simultaneously, the per capita TEPC and output remain constant. Under the assumption that the natural growth rate of population is n, the corresponding normal labor force, labor loss, and infected people all expand at a rate of n. Meanwhile, the proportion of the three groups in the total population remains invariable, and the total capital stock, the TEPC, and total output all increase at a rate of n. In conclusion, the above analysis demonstrates that, per capita output remains unaltered, and the growth rate of total output is uniquely determined by the population growth rate on the BGP.

References

Domar, E. D. (1946). Capital expansion, rate of growth, and employment. *Econometrica, Journal of the Econometric Society*, 137–147. `https://doi.org/10.2307/2225181`

Harrod, R. F. (1939). An essay in dynamic theory. *The Economic Journal*, *49*(193), 14–33. `https://doi.org/10.2307/2225181`
