# Supplementary material for: Public health events and economic growth in a neoclassical framework
Source: BMC Public Health. 2024 Jun 28;24:1724. doi: 10.1186/s12889-024-19106-4 (PMC11539698; doi:10.1186/s12889-024-19106-4)
Supplement: Supplementary file 3 — Supplementary Material 3. [file 12889_2024_19106_MOESM3_ESM.pdf]

Technological progress

In this section, we shall explore the production functions with technological progress under the background of RPC for a PHE. In general, considering a broader spectrum of technological progress types, the production function can be expressed as

$$Y(t) = A(t)^{\gamma} K(t)^{\alpha} L(t)^{1-\alpha}, \tag{C.1}$$

where $A(t)$ measures the level of technology, and it is assumed that $\dot{A}(t)/A(t) \equiv g$ is exogenously given, and γ is the technological progress parameter. Specifically, $\gamma = \alpha$ stands for capital-augmenting (Solow-neutral) technological progress, $\gamma = 1-\alpha$ for labor-augmenting (Harrod-neutral), $\gamma = 1$ for output-augmenting (Hicks-neutral).

Consequently, a model of economic growth incorporating technological progress is constructed by Equations (1), (5), (10) and (C.1). both sides of the equations are divided by $A(t)^{\gamma/(1-\alpha)}N(t)$ to get "per capita" forms, thus yielding the "per capita" production function

$$y(t) = k(t)^{\alpha} l(t)^{1-\alpha}, \tag{C.2}$$

where $\tilde{y}(t) = Y(t)/[A(t)^{\gamma/(1-\alpha)}N(t)]$ and $\tilde{k}(t) = K(t)/[A(t)^{\gamma/(1-\alpha)}N(t)]$ represent "per capita" output and capital, respectively, and $l(t) = L(t)/N(t)$ still represents the proportion of normal labor force to the total population.

The "per capita" capital dynamics will be rewritten as

$$\dot{\tilde{k}}(t) = (1-\beta_2)\tilde{k}(t)^{\alpha}\tilde{l}(t)^{1-\alpha} - [n + \gamma g/(1-\alpha) + \delta]\tilde{k}(t) - (1-\beta_2)\tilde{x}(t;\theta), \tag{C.3}$$

where $\tilde{x}(t;\theta) = X(t;\theta)/[A(t)^{\gamma/(1-\alpha)}N(t)]$ represents the "per capita" TEPC. It is noteworthy that the TEPC should also be increased in proportion to technological progress to offset the price effect when accounting for technological progress. Consequently, the subsequent analytical methods are analogous to those previously employed, and the corresponding results of Theorem 1-3 only need to add a tilde symbol above the relevant variables. The ensuing conclusions on the BGP are as follows: For any given PCI, "per capita" capital, TEPC and output remain unaltered. The total population, normal labor force, labor loss, and the infected people caused by the PHE all expand at a rate of n. And these categories of population to the total population remains constant. The total capital stock, TEPC and total output grow at a rate of $n + \gamma g/(1-\alpha)$. At this juncture, the growth rate of total output is determined by both the growth rate of population and technological progress.
